# Identifying Predictive Factors of Severity in Methamphetamine-Induced Psychosis Utilizing the Positive and Negative Syndrome Scale (PANSS)

**DOI:** 10.1192/bjo.2026.11191

**Published:** 2026-06-30

**Authors:** Sidra Rasheed, Imtiaz Ahmad Dogar, Iqra Ain Ali, Abdul Rehman Asgher, Hafiza Aneeqa Mustafa

**Affiliations:** Faisalabad Medical University, Faisalabad, Pakistan

## Abstract

**Aims:**

Methamphetamine-induced psychosis (MIP) is a severe psychiatric condition increasingly reported in low- and middle-income countries like Pakistan. It manifests with positive, negative, and general psychopathological symptoms, often mimicking primary psychotic disorders. Understanding predictors of severity is crucial for early intervention and prevention of chronicity. The aim is to quantify symptom severity in MIP using the Positive and Negative Syndrome Scale (PANSS) and identify demographic and substance-use-related predictors of higher PANSS scores.

**Methods:**

A cross-sectional analytical study was conducted over six months at DHQ/Allied II Hospital, Faisalabad. Ninety-three adults diagnosed with MIP as per DSM–5 criteria were assessed using PANSS. Data on demographics, age of onset, duration, frequency, and route of methamphetamine use were collected. Statistical analyses included correlation, ANOVA, and multivariate linear regression (p < 0.05).

**Results:**

The mean total PANSS score was 88.4 ± 14.2, indicating moderate-to-severe psychosis. Negative symptoms predominated (mean=24.5). Intravenous (IV) use, onset before age 25, and use duration > 2 years were independent predictors of higher total PANSS scores (β=12.8, 7.2, and 9.4, respectively; all p < 0.01). IV users had significantly higher scores than oral or smoking users (p < 0.001).

**Conclusion:**

MIP in this cohort is moderately severe, with negative symptoms notably elevated. Early initiation, prolonged use, and IV administration significantly worsen psychosis severity. These modifiable risk factors highlight targets for public health interventions and early clinical screening in substance use settings.

